# Progesterone Alleviates Endometriosis via Inhibition of Uterine Cell Proliferation, Inflammation and Angiogenesis in an Immunocompetent Mouse Model

**DOI:** 10.1371/journal.pone.0165347

**Published:** 2016-10-24

**Authors:** Yanfen Li, Malavika K. Adur, Athilakshmi Kannan, Juanmahel Davila, Yuechao Zhao, Romana A. Nowak, Milan K. Bagchi, Indrani C. Bagchi, Quanxi Li

**Affiliations:** 1 Department of Comparative Biosciences, University of Illinois at Urbana-Champaign, Urbana, Illinois, United States of America; 2 Department of Animal Science, University of Illinois at Urbana-Champaign, Urbana, Illinois, United States of America; 3 Department of Molecular & Integrative Physiology, University of Illinois at Urbana-Champaign, Urbana, Illinois, United States of America; Michigan State University, UNITED STATES

## Abstract

Endometriosis, defined as growth of the endometrial cells outside the uterus, is an inflammatory disorder that is associated with chronic pelvic pain and infertility in women of childbearing age. Although the estrogen-dependence of endometriosis is well known, the role of progesterone in development of this disease remains poorly understood. In this study, we developed a disease model in which endometriosis was induced in the peritoneal cavities of immunocompetent female mice, and maintained with exogenous estrogen. The endometriosis-like lesions that were identified at a variety of ectopic locations exhibited abundant blood supply and extensive adhesions. Histological examination revealed that these lesions had a well-organized endometrial architecture and fibrotic response, resembling those recovered from clinical patients. In addition, an extensive proliferation, inflammatory response, and loss of estrogen receptor alpha (ERα) and progesterone receptor (PR) expression were also observed in these lesions. Interestingly, administration of progesterone before, but not after, lesion induction suppressed lesion expansion and maintained ERα and PR expressions. These progesterone-pretreated lesions exhibited attenuation in KI67, CD31, and pro-inflammatory cytokine expression as well as macrophage infiltration, indicating that progesterone ameliorates endometriosis progression by inhibiting cell proliferation, inflammation and neovascularization. Our studies further showed that suppression of global DNA methylation by application of DNA methyltransferase inhibitor to female mice bearing ectopic lesions restrained lesion expansion and restored ERα and PR expression in eutopic endometrium and ectopic lesions. These results indicate that epigenetic regulation of target gene expression via DNA methylation contributes, at least in part, to progesterone resistance in endometriosis.

## Introduction

Endometriosis is a gynecological disorder defined as the growth of endometrial glands and stroma in extrauterine locations, primarily on the surfaces of the pelvic peritoneum, ovaries, and rectovaginal septum [1, 2]. It affects 6%–10% of women of reproductive age and the prevalence increases as high as 35%–50% in women experiencing chronic pelvic pain, and/or infertility that negatively affect their health and quality of life [3]. Although the etiology of endometriosis has not been defined, the widely accepted mechanism of the disease is the entry of the endometrial cell aggregates into the peritoneal cavity via the fallopian tubes, a process known as retrograde menstruation, where these endometrial fragments attach and invade into the peritoneal surfaces and grow into lesions at these ectopic sites [1, 2].

Prolonged exposure to estrogen (E_2_) is a major endocrine risk factor for endometriosis establishment and progression. Several lines of evidence have linked endometriosis with excessive E_2_ signaling in the ectopic tissues [4, 5]. The presence of an E_2_ biosynthesis machinery, including elevated levels of 17β-hydroxysteroid dehydrogenase-1 and aromatase, which produces excess E_2_ in ectopic lesions has been reported [6–8]. This local E_2_ activates estrogen receptors (ER) to stimulate mitotic activity and inflammatory responses. Hence, aromatase inhibitors and certain ER modulators have been proposed to alleviate the clinical symptoms of this disease [9–12]. There are two different forms of the ER, usually referred to as ERα and ERβ, and encoded by two separate genes, *Esr1* and *Esr2*, respectively. Early studies using ERα- or ERβ-null mice and selective estrogen receptor modulators revealed that both ERα and ERβ play essential roles in the establishment and development of ectopic lesions [9, 12, 13]. Interestingly, ample evidence indicates that ERβ is excessively expressed in the ectopic lesions when compared with normal endometrium [14].

In the normal endometrium, P_4_, via progesterone receptor (PR), counteracts E_2_-mediated action and exhibits anti-proliferative and anti-inflammatory roles [15, 16]. There are two PR isoforms, PRA and PRB, which are transcribed from a single gene (*Pgr*) with two alternative promoters. PRA and PRB differ only in that PRB contains an additional 164 amino acids at the N-terminus that are missing in PRA [17, 18]. Although the functional interaction between PR-A and PR-B is not required for reproductive activity [19], early studies indicated that loss of PR expression or perturbation of PR-mediated signaling is often associated with a hyperactive E_2_ action in the endometrium and development of female reproductive diseases including endometriosis [15, 20–24]. Hence, the antagonistic nature of P_4_ to E_2_ in endometrium empowers progestin as the first line of hormonal therapy for clinical treatment of endometriosis [25–28]. Unfortunately, the therapeutic efficacy and the beneficial effect of P_4_ on pathogenesis of endometriosis remain debatable. This is primarily due to the proliferative role of P_4_ in endometrial stromal cells that constitute a major cellular component in the ectopic lesions [29]. In addition, the efficacy of this hormone is only limited to a subset of patients. Protein analysis of PRA and PRB expression in the eutopic endometrium and the ectopic lesions of patients showed lower levels of PRA and PRB expression in the diseased tissues when compared with the endometrium of patients without disease [30–33]. Using experimental animals as disease models, early studies showed that PR and PR-mediated signaling components are often intact in the early stages of endometriosis. With disease progression, the overall level of PR expression and its targets decline in the eutopic endometrium and the ectopic lesions [34]. However, it remains unclear whether the P_4_ resistance in the endometrium predisposes women to endometriosis, and what the driving factors are that down regulate PR signaling in these disease tissues.

Although it is variable, the length of time between the onset of pain symptoms and the surgical diagnosis of endometriosis is estimated between 8 to 11 years in women from the UK and the USA [35]. Studies relying on clinically obtained disease samples preclude investigation of the early events in pathogenesis of this disease. Studies in immunocompromised mice aimed to circumvent host immune rejection and establish human disease tissues in mice. To delineate the time course of disease progression and investigate the roles of steroid hormones in pathogenesis of endometriosis, we adopted a syngeneic mouse model of endometriosis that recapitulates the retrograde mensuration in humans. Our studies revealed that PR and PR-mediated signaling are progressively lost in the developing ectopic lesions. Treatment of host female mice with P4 before endometriosis induction inhibits establishment and growth of ectopic lesions, primarily via suppression of proliferation, inflammation, and angiogenesis. Our studies further revealed that inhibition of DNA methylation in the host females ameliorates lesion growth and restored PR and PR-target gene expression indicating an involvement of epigenetic regulation in the pathogenesis of endometriosis.

## Materials and Methods

### Reagents

Progesterone (P8783) and 17-β-estradiol (E2758) were purchased from Sigma Chemical Co. (St. Louis, MO). 5-aza-2'-deoxycytidine (Decitabine, 3842) was purchased from Tocris Bioscience. Antibodies were purchased from Abcam (alpha smooth muscle actin/αSMA, ab5694; C-C Motif Chemokine Receptor 7/CCR7, ab103404; Cluster of Differentiation 68/CD68, ab955; CD206/mannose receptor, ab64693; Forkhead box P3/FOXP3, ab20034; and Platelet endothelial cell adhesion molecule/PECAM-1/CD31, ab28364), BD PharMingen (Ki67, 550609), Cell Signaling (Vimentin/VIM, 5714S), Dako (PR, A0098), eBioscience (EGF-like module-containing mucin-like hormone receptor-like 1, EMR1 (F4/80),12–4801; RAR-related orphan receptor gamma/RORγ(t), 14–6988), Leica Biosystems (ERα, NCL-L-ER-6F11), Developmental Studies Hybridoma Bank (Cytokeratin 11/KRT11/CK11, TROMA-I-s; Jackson ImmunoResearch Laboratories (Alexa Fluor® 488 AffiniPure Donkey Anti-Rabbit IgG (H+L), Code: 711-545-152; Rhodamine Red™-X (RRX) AffiniPure Donkey Anti-Mouse IgG (H+L), 715-295-150), Novus Biologicals (Cysteine-rich angiogenic inducer 61/CYR61/CCN1, NB100-356), and Santa Cruz Biotechnology (Heart And Neural Crest Derivatives Expressed 2/dHand/HAND2, sc-9409), respectively (Table A in S1 File).

### Ovariectomy and mouse model of endometriosis

All experiments involving animals were approved by the University of Illinois Institutional Animal Care and Use Committee and conducted in accordance with National Institutes of Health standards for the use and care of animals. Animals were maintained at the animal facility of College of Veterinary Medicine, University of Illinois at Urbana-Champaign in accordance with the applicable portions of the Animal Welfare Act and the guidelines prescribed in the DHHS publication, “Guide for the Care and Use of Laboratory Animals”. CD-1 female mice were purchased from Charles River. Mice were kept in a standard light controlled animal room (12 h day light) at 23–25˚C in polypropylene cages and provided with a rodent diet with minimal natural phytoestrogens and water *ad libitum*.

Ovariectomy: Adult mice (6–8 weeks of age) were anesthetized using a cocktail of Ketamine/Xylazine (87mg/kg; 15mg/kg) by intraperitoneal injection. The initial site for the incisions for mice was dorsal in the midline (approximately 1 cm), then by a similar incision lateral to the midline through the muscle (0.5 cm). Following the muscle incision, ovaries were located and excised, followed by closure of the muscular incisions with sterile resorbable suture and skin incisions with wound clips. Buprenorphine (0.05 mg/kg, subcutaneously) was used as analgesia to minimize discomfort, distress, and pain before and after surgery. Wound clips were removed 10–14 days post-operation.

**Fig 1:**
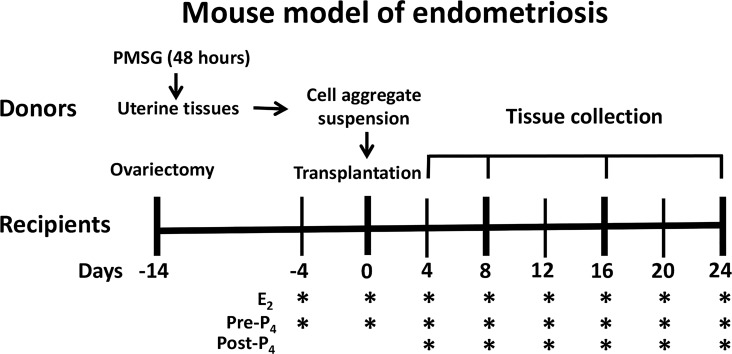
Immunocompetent mouse model of endometriosis. 6–8 weeks-old CD1 female mice were primed with PMSG for 48 hours. Uterine tissues were then harvested and minced into tiny cell aggregates after myometrial removal. Female mice with the same genetic background were subjected to ovariectomy and served as recipients. 2 weeks after surgery, equal volumes of uterine cell aggregate suspension were transferred into the peritoneal cavities of recipients. Endometriosis was maintained by subcutaneous administration of 100 ng of E_2_ once every 4 days until tissue collection. For the studies of the role of P_4_ in endometriosis, 1 mg of P_4_ (pre-P_4_) was administrated along with E_2_ beginning at 4 days before transplantation. For P4-resistance experiments (Post-P_4_), 1 mg of P_4_ was administration along with E2 beginning at 4 days after transplantation. The ectopic lesions were assessed under a dissecting microscope at different days after endometrial cell transplantation (n = 6 per treatment group).

Mouse model of endometriosis: Donor female mice were primed with pregnant mare serum gonadotropin (PMSG) for 48 hours to stimulate growth of the uterus. At the time of tissue transplantation, uterine tissues were harvested and minced into smaller cell aggregates in warm Hank’s buffered saline after careful peeling off myometrial layers. Syngeneic females served as recipients and were subjected to ovariectomy. 14 days after surgery, equal volumes of uterine cell suspension were transferred into the peritoneal cavities of the recipients at a ratio of 1 donor to 2 recipients. A gentle massage was given to disperse the tissue fragments. 2 weeks after surgery and 4 days before endometriosis induction, the recipients were injected subcutaneously with a physiological dose of E_2_ (100ng/mouse), then once every 4 days, to maintain endometriosis progression. For P_4_ pre-treatment experiments (Pre-P_4_), P_4_ (1mg/mouse) was administrated along with E_2_ beginning at 4 days before endometriosis induction. For P_4_-post-treatment experiments (Post-P_4_), P_4_ was administrated along with E_2_ beginning at 4 days after endometriosis induction. For DNA methylation experiments, Decitabine (0.5 mg/kg/day) was injected intraperitoneally once every other day beginning at 4 days after endometriosis induction until tissue collection. Recipients were euthanized by different days after transplantation (n = 6 per treatment group), the number, location, and sizes of ectopic lesions were assessed under a dissecting microscope, followed by histological evaluation (Fig 1).

### RNA isolation and quantitative real-time PCR analysis (qPCR)

Uterine tissue collection, RNA purification, cDNA synthesis, and qPCR were performed as described previously [36]. Primer sequences corresponding to specific target genes are listed in Table B in S1 File. *36B4*, encoding an acidic ribosomal phosphoprotein, served as an internal control. The mean ∆Ct was calculated from individual ∆Ct values obtained from a minimum of three replicates. ∆∆Ct was calculated as the difference between the mean ∆Ct values of the experimental and control samples. The fold change of gene expression in each sample relative to a control was computed as 2^–∆∆Ct^. The relative gene expression level was expressed as the average fold change ± SEM from at least three independent experiments.

### Histological evaluation

Sections of paraffin-embedded uterine tissues and ectopic lesions were deparaffinized, rehydrated in xylene and a series of graded ethanol, and subjected to histological evaluation. For Masson’s trichrome staining, sections were first incubated with Weigert's hematoxylin solution, followed by biebrich scarlet solution, phosphomolybdic acid solution and aniline blue solution to stain nuclei, plasma, and collagen, respectively. Immunohistochemistry (IHC) and Immunofluorescence (IF) analysis were performed as described previously [22]. Briefly, sections were subjected to antigen retrieval with citrate buffer (pH 6.0), and then incubated with the corresponding primary antibodies, respectively. For IHC, sections were incubated with the horseradish peroxidase-labeled Avidin-Biotin system (Vector Laboratories, Burlingame, CA) and color was developed by incubation briefly with AEC chromogen (3-amino-9-ethylcarbazole) substrate. Sections were counterstained with hematoxylin and mounted. Red color deposits indicate the sites of immunostaining. For IF, after primary antibody incubation the sections were labeled with Alexa Fluor 488- or Rhodamine Red-conjugated donkey serum anti-mouse/rabbit IgG (H+L), receptively, and then mounted with medium containing DAPI. The sections were examined under the microscope (Olympus BX51), photographed. Images with the same magnification were then analyzed by ImageJ software (ImageJ 1.49, NIH, USA) as described previously [37]. The number of positive cells and the staining intensities were quantified by comparing the average number of positively stained cells to the total number of cells in the demarcated areas from three independent samples.

### DNA methylation analysis

Genomic DNA samples were purified from the ectopic lesions and donor’s endometrium following the protocol of Allprep DNA/RNA purification kit (Qiagen), then digested with Methylation-sensitive, Methylation-dependent, or both enzymes, respectively. Equal amounts of digested DNAs were then subjected to qPCR following the EpiTect Methyl II Assay protocol (Qiagen) using specific primers flanking the known/predicted “CG” islands in the promoter regions of mouse *Pgr* (EPMM111296-1A) and *Hoxa10* (EPMM109276-1A), respectively. The methylated or non-methylated lymph node genomic DNA (New England Biolabs) served as positive or negative controls. Levels of DNA methylation were determined by the average ΔCt values obtained from qPCR amplification of mock-digested, methylation-sensitive enzyme-digested, methylation-dependent enzyme-digested and double-digested DNA samples following product protocol. The fold induction of DNA methylation in the ectopic lesions (D16) compared to that in the donor’s endometrium (D0) was determined.

### Statistical analysis

All of the numerical values were obtained from at least three independent samples and were analyzed by One-way ANOVA followed by Dunnett’s post hoc test when comparisons were made between a control group and more than one experimental group, or by Student’s *t*-test for single comparison (GraphPad Prism 5.0, GraphPad Software, Inc., San Diego, CA). Data are expressed as mean ± SEM. Statistical significance is defined as *p* < 0.05.

## Results

### P4 alleviates establishment and progression of ectopic lesions in mouse model of endometriosis

**Fig 2:**
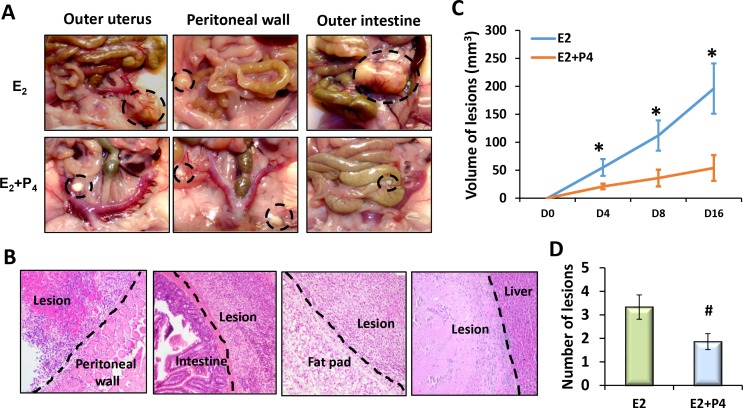
P_4_ alleviates E_2_-dependent establishment and growth of ectopic lesions. Ectopic lesions were established in the peritoneal cavities of immunocompetent female mice and treated with E_2_ or P_4_ along with E_2_ as described in Fig 1 (N = 6). (A) Locations of ectopic lesions (Dashed circles). (B) Histology of ectopic lesions. Dashed lines indicate the contact sites of the ectopic lesions with adjacent peritoneum. Representative images of ectopic lesions harvested at four weeks after induction are shown (40X). (C) Time-course progression of ectopic lesions. The average volumes of the ectopic lesions at the indicated time points are shown. (D) The average numbers of ectopic lesions identified in each treatment group are shown. The numerical values were analyzed by One-way ANOVA followed by Dunnett’s post hoc test and expressed as mean ± SEM. Statistical significance is defined as ^#^: p < 0.05, *: p<0.01.

To investigate the molecular mechanisms underlying the time course of disease progression and steroid hormone regulation in pathogenesis of endometriosis, we adopted a syngeneic mouse model of endometriosis with minor modification. This model recapitulates the retrograde menstruation in human [13]. In this model, tiny endometrial cell aggregates, without the myometrium, were inoculated into the abdominal cavities of the syngeneic females to allow random attachment and growth (Fig 1). The recipient mice were ovariectomized, and then treated with E_2_ or E_2_ and P_4_ subcutaneously once every 4 days beginning at 4 days before lesion induction (Pre-P_4_ treatment). The number, location, and size of the ectopic lesions were assessed in these mice under a dissecting microscope, followed by histological evaluation. The endometrial implants were established as early as 24 hours after transplantation and eventually developed into endometriosis-like lesions at a variety of places, primarily on the surfaces of the parietal peritoneum, visceral peritoneum and mesentery covering the uterus, gut, liver, kidney, and intestines, and abdominal fat pads (Fig 2A and 2B). The volumes of these ectopic lesions expanded with the time of progression (Fig 2C). Interestingly, E_2_-treated recipients had 2–5 large, light yellow color lesions with abundant blood vessels and extensive adhesions after four weeks of induction. In contrast, 1–3 small, white color and nonvascular lesions with loose attachment were seen in the E_2_ plus P_4_-treated group (Fig 2C and 2D). Hence, this syngeneic mouse model of endometriosis provides unique opportunity to study the steroid hormone-regulated molecular mechanisms that are associated with the establishment and progression of endometriosis.

### P_4_ restricts expansion of the ectopic lesions by inhibiting endometrial cell proliferation and neovascularization

**Fig 3:**
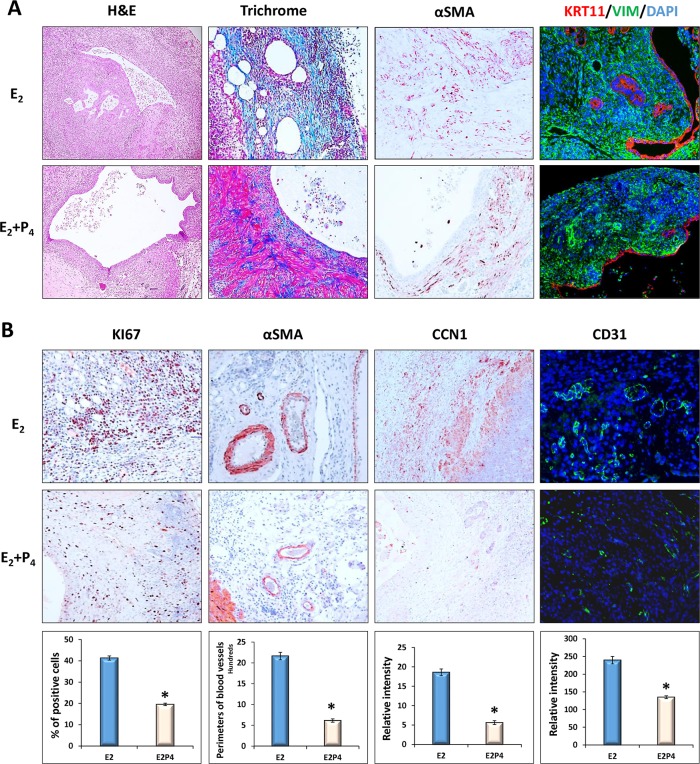
P_4_ inhibits E_2_-dependent cell proliferation and angiogenesis in ectopic lesions. Sections of the ectopic lesions collected from E_2_ or E_2_ plus P_4_-treated recipients (D16, n = 6) were subjected to histological examination. (A) Representative images (20X) showing H&E and Trichrome staining, or IHC analysis using antibody against myofibroblast biomarker αSMA, uterine epithelial biomarker KRT11, or uterine stromal biomarker VIM, respectively. (B) Representative images (20X) showing IHC analysis using antibodies against cell proliferation biomarker KI67, smooth muscle biomarker αSMA, endothelial cells CD31, or an angiogenetic regulator CCN1, respectively. The numbers of KI67-positive cells, the perimeters of the supporting blood vessels, and the immunostaining intensities of CCN1 and CD31 were analyzed by ImageJ software. The numerical values were analyzed by One-way ANOVA followed by Dunnett’s post hoc test and expressed as mean ± SEM. Statistical significance is defined as #: p < 0.05, *: p<0.01.

To further characterize the ectopic lesions obtained from our mouse model of endometriosis, lesion sections were subjected to histological examination by H&E and trichrome staining, or IHC analysis using antibodies against epithelial and stromal cell biomarkers cytokeratin 11 (KRT11) or Vimentin (VIM), respectively. As shown in Fig 3A, these lesions had a well-organized stromal and glandular architecture with glandular cysts filled with inflammatory cells and luminal epithelial cell debris. In addition, an extensive fibrotic response was also observed in the stromal cell compartment as indicated by the staining of the collagen-rich connective tissue (Trichrome) and a myofibroblast and smooth muscle biomarker, αSMA [38].

Ectopic lesion expansion depends on endometrial cell proliferation and neovascularization. To investigate the role of P_4_ in the developmental process of endometriosis, cell mitotic activity and angiogenesis were further compared in the ectopic lesions collected from host females treated with E_2_ or P_4_ along with E_2_, respectively. As shown in Fig 3B, E_2_-treated lesions exhibited extensive mitotic activity with large supporting blood vessels established at the periphery of lesions and the junctional region near the host peritoneum. Interestingly, in the E_2_ plus P_4_-treated lesions there was a significant reduction in uterine cell proliferation (KI67), perimeters of supporting blood vessels as well as expression of CD31 (an endothelial cell marker also known as PECAM-1) and CCN1/Cyr61, a critical angiogenic regulator in pathogenesis of endometriosis [39]. Collectively, these results indicated that P_4_ attenuates E_2_-dependent establishment and growth of ectopic lesions, most likely by inhibition of endometrial cell proliferation and angiogenesis.

### P_4_ suppresses E_2_-dependent inflammatory responses in the ectopic lesions

Immune cell infiltration is a hallmark of endometriosis [40]. Macrophages are particularly interesting in this disease, primarily due to their abundance as well as scavenging activity of misplaced/damaged cells in the peritoneal cavity. Ample evidence exists to indicate that macrophage function can be polarized to a wide spectrum of phenotypes depending upon the inflammatory stimuli in the microenvironment [41]. The inflammatory phenotype M1 is mainly responsible for clearance of infectious pathogens and damaged/apoptotic cells by producing pro-inflammatory factors (e.g., IL-6, IL-1b, IL-8, NOS, PGE2 and TNFα) [41]. The anti-inflammatory/pro-angiogenic phenotype M2, however, is predominantly involved in immunosuppression and tissue repair by producing factors such as IGF1, TGFβ, IL-10, and VEGF [41]. Early studies indicated that both macrophage phenotypes are present in the peritoneal fluid and the ectopic lesions of patients [42]. To investigate the impact of P_4_ on the inflammatory responses in endometriosis, we first examined infiltration and activation of peritoneal macrophages in response to endometrial cells by IHC or IF analysis using antibodies against the pan-macrophage biomarker (F4/80), M1 (CCR7), or M2 (CD206), respectively [41]. As shown in S1 and S2 Figs, in response to endometrial cell stimulation the number of peritoneal cells increased rapidly, but equally, in both E_2_ and E_2_ plus P_4_-treated female mice, then declined by day 16 and returned to basal levels by 24 days after lesion induction. When macrophage biomarkers were examined in these cells, we found that in the absence of endometrial cells more than half of the peritoneal cells were large, M2-like naïve resident macrophages that are positive for F4/80 and CD206 with a low level of CCR7 expression (CD206^hi^/CCR7^lo^/F4/80^hi^). These cells, however, rapidly disappeared from the peritoneal cavity following endometrial inoculation, and were replaced with abundant small monocyte-derived inflammatory macrophages exhibiting a high level of CCR7 with low level of F4/80 and CD206 expression (CD206^lo^/CCR7^hi^/F4/80^lo^). As disease progression continued, the CD206 level gradually increased and CCR7 level gradually decreased, until eventually all of the peritoneal macrophages acquired a F4/80^hi^/ CD206^hi^/CCR7^lo^ phenotype. This phenomenon is similar to that observed during peritonitis indicating activation and M1 to M2 transition of macrophages during progression of endometriosis [43]. Surprising, there was no apparent difference in the numbers of M1 or M2 macrophages in E_2_- and E_2_ plus P_4_-treated groups when examined 16 days after endometriosis induction (S2 Fig).

**Fig 4:**
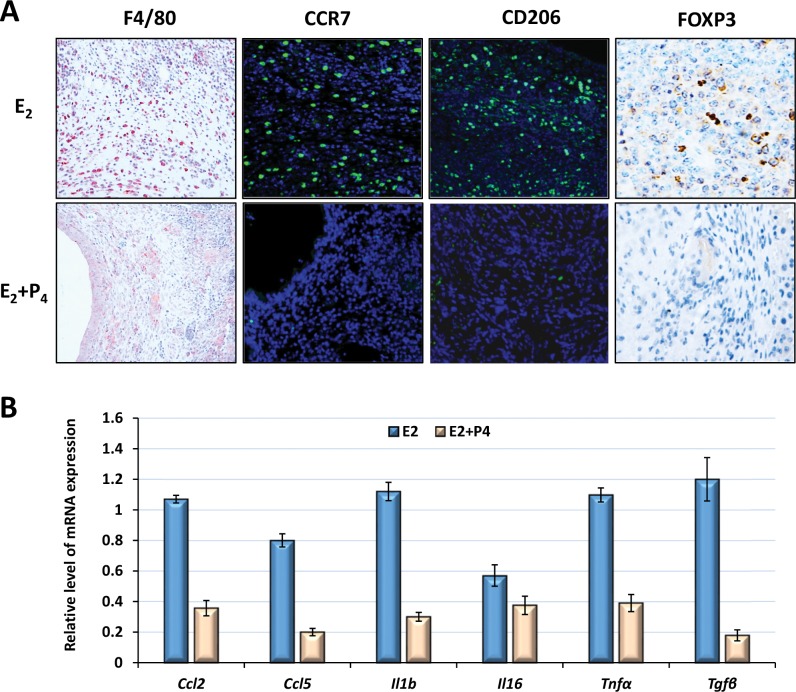
P_4_ suppresses E_2_-dependent inflammatory responses in the ectopic lesions. Ectopic lesions were harvested from E_2_ or E_2_ plus P_4_-treated recipients (n = 6) by 16 days after induction. (A) Representative images (20X) showing IHC analysis using antibodies against pan-macrophage biomarker (F4/80), inflammatory M1 (CCR7), anti-inflammatory M2 (CD206) macrophages or Treg cell biomarker (FOXP3), respectively. (B) The relative level of mRNA expression corresponding to *Ccl2*, *Ccl5*, *Il1b*, *Il6*, *Tnfa*, and *Tgfb* was analyzed by qPCR after moralization to the internal control gene, *36B4*. The numerical values were analyzed by One-way ANOVA followed by Dunnett’s post hoc test and expressed as mean ± SEM. Statistical significance is defined as #: p < 0.05, *: p<0.01.

We next investigated activation and infiltration of macrophages in the ectopic lesions. As shown in Fig 4A, abundant cells that are positive for CD206, CCR7, or F4/80 infiltrated into E2-treated ectopic lesions (D8). We also examined expression of macrophage-derived pro-inflammatory cytokines (IL-6 and IL-1b), chemotactic factors (CCL2 and CCL5) and pro-angiogenic growth factors (TNFα and TGFβ) in these lesions by qPCR analysis. Our results showed that all of these factors were rapidly induced in the ectopic lesions. While *Ccl2*, *Il6*, and *Il1b* expression peaked by 4 days after lesion induction, expression levels of *Ccl5*, *Tgfb*, and *Tnfa* were sustained until day 16 (S3 Fig). Interestingly, the number of activated macrophages and expression levels of the inflammatory factors diminished dramatically in the ectopic lesions in response to P_4_ administration (Fig 4). We also examined expression of B cell surface marker CD45R, T Regulatory cell (Treg) marker FOXP3, and T helper 17 cell (Th17) marker RORγ in these lesions. No apparent difference in B cell populations was observed between E_2_ and E_2_ plus P_4_-treated ectopic lesions. Treg cell populations were decreased in response to P_4_ stimulation, whereas Th17 cells were not observed in either group of lesions (Fig 4A). Collectively, these results indicate that P_4_ regulates E_2_-dependent inflammatory responses in endometriosis, primarily by suppressing production of the pro-inflammatory cytokines and infiltration of immune cells in the ectopic lesions.

### Progressive loss of ERα/PR-mediated signaling in the ectopic lesions leads to resistance to P_4_ therapy

**Fig 5:**
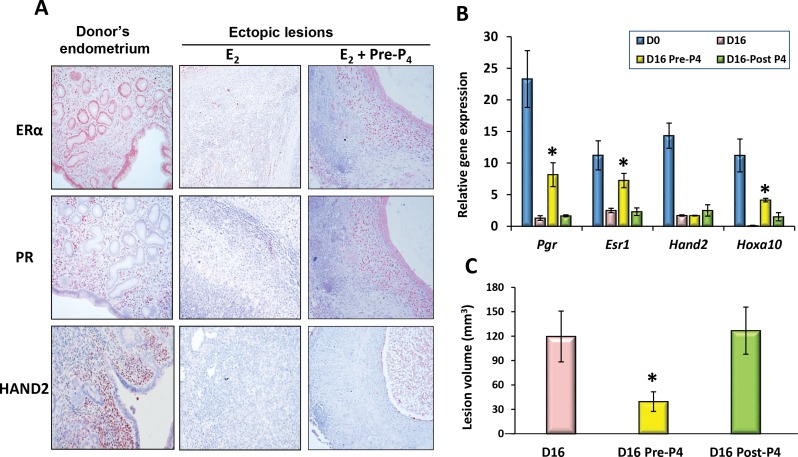
Loss of ERα/PR-mediated signaling contributes to P_4_-resistance in this mouse model of endometriosis. Endometriosis was induced and maintained with E_2_ as described in Fig 1. The host females were then treated with P_4_ beginning at 4 days before (Pre-) or 4 days after (Post-) endometrial cell transplantation until tissue collection (n = 6). Donor uterine tissue (D0) and ectopic lesions were subjected to IHC analysis (A) for ERα, PR and HAND2 protein expression (20X) or qPCR analysis (B) to assess expression level of mRNA corresponding to *Esr1*, *Pgr*, *Hand2*, and *Hoxa10*, respectively. (C) Lesion volumes were quantitated by 16 days after induction. The numerical values were analyzed by One-way ANOVA followed by Dunnett’s post hoc test and expressed as mean ± SEM. Statistical significance is defined as #: p < 0.05, *: p<0.01.

Several lines of evidence now suggest that endometrial stromal cells, collected from the eutopic endometrium and the ectopic lesions of patients, exhibit insufficient response to P_4_ stimulation. To investigate the time-course progression of PR-mediated signaling in endometriosis, the ectopic lesions collected from our mouse model of endometriosis were first subjected to qPCR to analyze gene expression levels of PR-signaling components. Interestingly, our results showed a progressive down-regulation of mRNA expression corresponding to *Esr1*, *Pgr*, and PGR-stromal targets *Hand2* and *Hoxa-10* with time of disease progression. In contrast, *Esr2* mRNA gradually increased in these lesions (S4 Fig). Immunostaining of these lesions also showed a sharp decline in the protein levels of ERα, PR and HAND2 when compared to the donor uterine tissues (Fig 5A, *middle vs left panels*). Interestingly, *Esr1*, *Pgr*, and *Hoxa10* expression were sustained in the ectopic lesions when P_4_ was administrated to the host females before lesion induction, while *Hand2* expression remained suppressed (Pre-P_4_ in Fig 5A and 5B). In contrast, the P_4_-mediated inhibitory effect was not seen in host females when P_4_ treatment was started 4 days after lesion induction (Post-P_4_ in Fig 5B and 5C). These results further support the notion that loss of PR-mediated signaling during disease progression contributes to the increased susceptibility to P_4_ resistance in endometriosis patients.

### Epigenetic regulation of PR-mediated signaling molecules via DNA methylation is involved in pathogenesis of endometriosis

**Fig 6:**
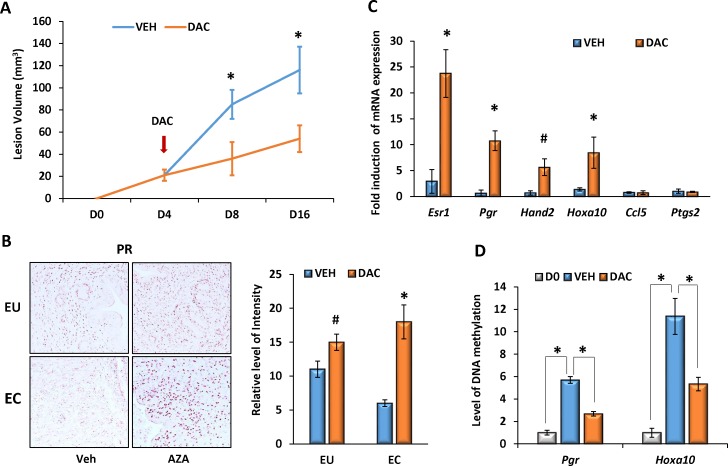
DNA methylation is involved in regulation of PR-signaling in endometriosis. Endometriosis was induced in female mice and maintained with E_2_. Beginning at 4 days after induction, host females were treated with either vehicle (VEH) or 0.5 mg/kg of Decitabine (DAC) intraperitoneally, once every other day until tissues collection (n = 6). (A) Lesion volumes were measured at 8 and 16 days after induction. (B) The eutopic endometrium (EU) and the ectopic lesions (EC) were subjected to IHC analysis for PGR protein expression (20X). Representative images from each group are shown. The relative intensities of PR staining were analyzed by ImageJ software. (C) qPCR was employed to analyze relative levels of gene expression in the ectopic lesions (D16) treated with VEH or DAC. (D) DNA methylation levels of *Hoxa10* and *Pgr* promoters were assessed by qPCR following digestion of genomic DNA with methylation-specific enzymes. The numerical values were analyzed by One-way ANOVA followed by Dunnett’s post hoc test and expressed as mean ± SEM. Statistical significance is defined as #: p < 0.05, *: p<0.01.

It is well known that epigenetic regulation of target gene expression is involved in pathogenesis of many female reproductive diseases [44–50]. To explore the potential role of DNA methylation in the development of endometriosis, we utilized a DNA methyltransferase (DNMT) inhibitor Decitabine (DAC, 5-aza-2'-deoxycytidine), an analogue of deoxycytidine that can incorporate into DNA strands and cause DNA demethylation [51]. Endometriosis was induced and maintained in E_2_-treated female mice. Beginning at day 4 after induction, host females were treated with either vehicle (VEH) or 0.5 mg/kg of DAC, intraperitoneally, once every other day, until tissue collection. Interestingly, DAC significantly inhibited growth of the ectopic lesions by 8 and 16 days after lesion induction (Fig 6A). In addition, administration of this inhibitor markedly increased expression levels of PR protein as well as mRNA corresponding to *Esr1*, *Pgr*, *Hand2*, and *Hoxa10*, but not for *Ccl5* and *Ptgs2* in the eutopic endometrium and the ectopic lesions (Fig 6B and 6C). We further compared the levels of DNA methylation in promoter regions of *Hoxa10* and *Pgr* in the donor uterine tissues and the ectopic tissues (D16) treated with or without inhibitor. Equal amounts of the genomic DNA were digested with DNA methylation-dependent or sensitive enzymes, and the relative level of DNA methylation was determined by qPCR. Compared to the donor’s endometrium, the levels of DNA methylation in the promoters of *Hoxa10* and *Pgr* were markedly enhanced in the ectopic lesions and this increase in DNA methylation was suppressed significantly by the DNMT inhibitor treatment (Fig 6D), indicating that DNA methylation is involved, at least in part, in the pathogenesis of endometriosis by affecting target gene expression.

## Discussion

Progestin treatment is considered as one of the standard hormonal therapies for alleviating clinical symptoms and preventing recurrence of endometriosis [25–28]. However, the therapeutic efficacy of this treatment is limited to only a subset of patients, impeding routine clinical application. The P_4_-resistance is primarily due to the low level of PR expression in the eutopic endometrium and the ectopic lesions of patients [30–34]. Consistently, in our mouse model of endometriosis we observed that expression of ERα, PR, and PR-stromal targets progressively declined in the diseased tissues, leading to unresponsiveness to the subsequent P_4_ treatment. A similar observation was also reported in the nonhuman primate model of endometriosis in which expression levels of PR and its downstream targets were diminished in the eutopic endometrium and the ectopic lesions with disease progression [34]. Interestingly, P_4_ is able to ameliorate lesion outgrowth and maintain ERα and PR expression when this hormone was administrated early prior to lesion induction. These results clearly indicate that the loss of PR-mediated signaling components is a major causal factor for the P_4_ resistance that exists in females with endometriosis. Hence, PR and PR-downstream targets may serve as useful biomarkers to predict efficacy of hormonal therapy and recurrence of endometriosis.

The underlying mechanisms involved in the regulation of the molecules that are critical for steroid responsiveness in endometriosis remain poorly understood. Early studies showed that the ERβ expression level in endometriosis is >100 times higher than that in normal endometrial tissues [14]. Since ERβ can bind directly to the ERα promoter region to repress ERα gene expression, it was believed that the high ratio of ERβ-to-ERα in the ectopic endometrial cells would result in suppression of ERα/PR expression, thus contributing to P_4_ resistance [14]. P_4_ responsiveness may also be impaired by PR-coregulators and/or their downstream effectors, e.g. FKBP52, HIC-5/ARA55, KLF9/13, HOXA10, HAND2 or FOXO1, that play critical roles in PR-mediated endometrial functions, but are aberrantly expressed in eutopic endometrium and ectopic lesions of endometriosis [21, 23, 52, 53]. A recent study also showed that inhibition of the v-akt murine thymoma viral oncogene homolog (AKT)-mediated signaling pathway impaired development of endometriosis and restored PR and FOXO1 expression in ectopic lesions [54].

Ample evidence suggests that epigenetic regulation including DNA methylation is involved in pathogenesis of many female reproductive diseases including endometriosis [55–57]. Early studies showed that the promoter sequences of ERα and ERβ (*Esr1* and *Esr2*), PRs (*Pgr-*A and B), as well as PR-targets *Hoxa10*, *Gata2/6*, and *Hand2* are susceptible to DNA methyltransferases, leading to an aberrant expression of these molecules in endometrial diseases [44–50]. Many laboratories are currently investigating genome-wide alteration of DNA methylation in clinically obtained samples [50, 58–60], however the primary drivers for these epigenetic aberrations remain unclear. Several lines of evidence have suggested that induction of pelvic endometriosis is sufficient to trigger long-lasting changes of target gene expression in the eutopic endometrium [47, 61]. Since chronic inflammation is closely involved in disease progression of endometriosis [40], and the inflammatory factors produced by the activated immune cells, notably macrophages and neutrophils, are important epigenetic modifiers that provoke widespread changes in the chromatin landscape of many cell types [62–67], it is plausible to envisage a mechanism in which paracrine inflammatory factors produced by ectopic endometrial cells and activated immune cells act on the eutopic endometrium and the ectopic lesions, to regulate target gene expression through epigenetic mechanisms. Indeed, our current studies showed that DNA methylation of the CG islands in *Pgr* and *Hoxa10* promoters was enhanced in the ectopic lesions in comparison to the normal endometrium. Inhibition of genome-wide DNA methylation in female mice restrained lesion expansion and partially restored target gene expression. These results indicate that epigenetic regulation of target gene expression via DNA methylation contributes, at least in part, to P_4_-resistance in endometriosis.

E_2_ and P_4_ exert opposing effects in controlling the inflammatory responses in the endometrium. Specifically, E_2_ enhances inflammation by directly promoting production of cytokines, including TNFα, TGFβ, MMP7, RANTES, IL6, VEGFs, and activation of NF-κB-mediated inflammatory pathways, whereas P_4_ inhibits them [40, 68, 69]. Hence, a hyperactive E_2_ action and sustained inflammatory response were frequently observed in the uterine tissues of female mice lacking PR or PR-mediated signaling molecules [20, 70]. In the ectopic lesions, both ERα and ERβ contribute to the E_2_-induced inflammatory responses. Recent studies by the Katzenellenbogen group showed that inhibition of ERβ activity by ERβ-selective antagonists in ectopic lesions suppressed inflammation and lesion growth [9]. Studies by O’Malley *et al* also showed that ERβ could enhance inflammation and endometriosis progression by interacting with components of the cytoplasmic inflammasome to increase interleukin-1β [12]. Although there is no evidence currently available to show that PR could potentially interact with ERβ directly, it is possible that ERβ may impair PR-mediated signaling in the ectopic lesions indirectly through SRC1, a PR coactivator [71]. In endometriosis, both inflammatory (M1) and anti-inflammatory (M2) macrophages are present in the peritoneal fluid and ectopic lesions. Adequate evidence suggests that both ERα and PR are present in peritoneal macrophages, and E_2_ and P_4_ could influence the inflammatory functions of these cells [72]. Interestingly, our studies revealed that P_4_ exhibits minimal effects on infiltration and activation of peritoneal macrophage in response to endometrial cells, however we cannot exclude the possibility that P_4_ exerts an immunomodulatory action by controlling production of pro-inflammatory factors and/or pro-angiogenic growth factors in these macrophages [73–75].

In summary, using a mouse model of endometriosis our studies unraveled a potential mechanism underlying P_4_-resistance that is associated with the loss of PR-mediated signaling in endometriosis. Our studies have clearly shown that early P_4_ treatment, when PR is present, could preserve steroid responsiveness and ameliorate E_2_-dependent disease progression. On the other hand, P_4_ has a minimal effect on disease progression when this hormone is administrated along with E_2_ at a later stage when PR is absent. Deciphering the molecular mechanisms by which PR-mediated signaling is aberrantly regulated in the eutopic endometrium and ectopic lesions of patients will provide mechanistic insights into the pathogenesis of endometriosis, and lead to identification of novel prognostic biomarkers to predict the efficacy of hormonal therapy and recurrence of endometriosis.

## Supporting Information

S1 Fig Peritoneal macrophages are activated in this mouse model of endometriosis. Endometriosis was induced in immunocompetent host females and maintained with E_2_. Peritoneal cells were harvested at the indicated time points and subjected to double Immunofluorescent labeling for CD206 or CCR7 with F4/80, respectively. Representative images at each time point are shown (top panels, 20X). The percentages of positive cell numbers for CD206, CCR7, CCR7^+^/F4/80^+^, or CD206^+^/F4/80^+^ are shown (bottom panel). The numerical values were analyzed by One-way ANOVA followed by Dunnett’s post hoc test and expressed as mean ± SEM (n = 5). *p < 0.01 verse D0.
(TIF)

S2 Fig P_4_ exhibits minimal effects on peritoneal macrophages. Ectopic lesions were induced in E_2_-, or E_2_ plus P_4_-treated immunocompetent host females. The total number of exuded peritoneal cells were counted (**A**) and subjected to double immunofluorescent labeling for CD206 or CCR7 with F4/80 (**B**). The average percentages of cell numbers for CCR7^+^/F4/80^+^ or CD206^+^/F4/80^+^ are shown. The numerical values were analyzed by One-way ANOVA followed by Dunnett’s post hoc test and expressed as mean ± SEM (n = 5). Statistical significance is defined as #: p < 0.05, *: p<0.01 verse D0.
(TIF)

S3 Fig Rapid induction of pro-inflammatory cytokines in ectopic lesions. Endometriosis was induced in immunocompetent female mice and maintained with E_2_. The ectopic lesions were harvested on days 0, 4, 8, and 16 after induction and subjected to qPCR analysis to assess the relative level of gene expression corresponding to *Ccl2*, *Ccl5*, *Il1b*, *Il6*, *Tnfα*, and *Tgfβ*, respectively. The numerical values were analyzed by One-way ANOVA followed by Dunnett’s post hoc test and expressed as mean ± SEM (n = 6). Statistical significance is defined as #: p < 0.05, *: p<0.01 verse D0.
(TIF)

S4 Fig Progressive declines in expression of ERα/PR-mediated signaling components in the developing ectopic lesions. Endometriosis was induced in immunocompetent female mice and maintained with E_2_. The ectopic lesions were harvested on Days 0, 4, 8, and 16 after induction (N = 6) and subjected to qPCR analysis to assess the relative level of gene expression corresponding to *Esr1*, *Pgr*, *Hand2*, and *Hoxa10* (upper panel), or *Esr2* (Lower panel), respectively. The numerical values were analyzed by One-way ANOVA followed by Dunnett’s post hoc test and expressed as mean ± SEM. Statistical significance is defined as #: p < 0.05, *: p<0.01 verse D0.
(TIF)

S1 File Antibodies and Primers used in this study. (PDF)
