# Supplementary figures and images for: Progesterone Alleviates Endometriosis via Inhibition of Uterine Cell Proliferation, Inflammation and Angiogenesis in an Immunocompetent Mouse Model
Source: PLoS One. 2016 Oct 24;11(10):e0165347. doi: 10.1371/journal.pone.0165347 (PMC5077092; doi:10.1371/journal.pone.0165347)

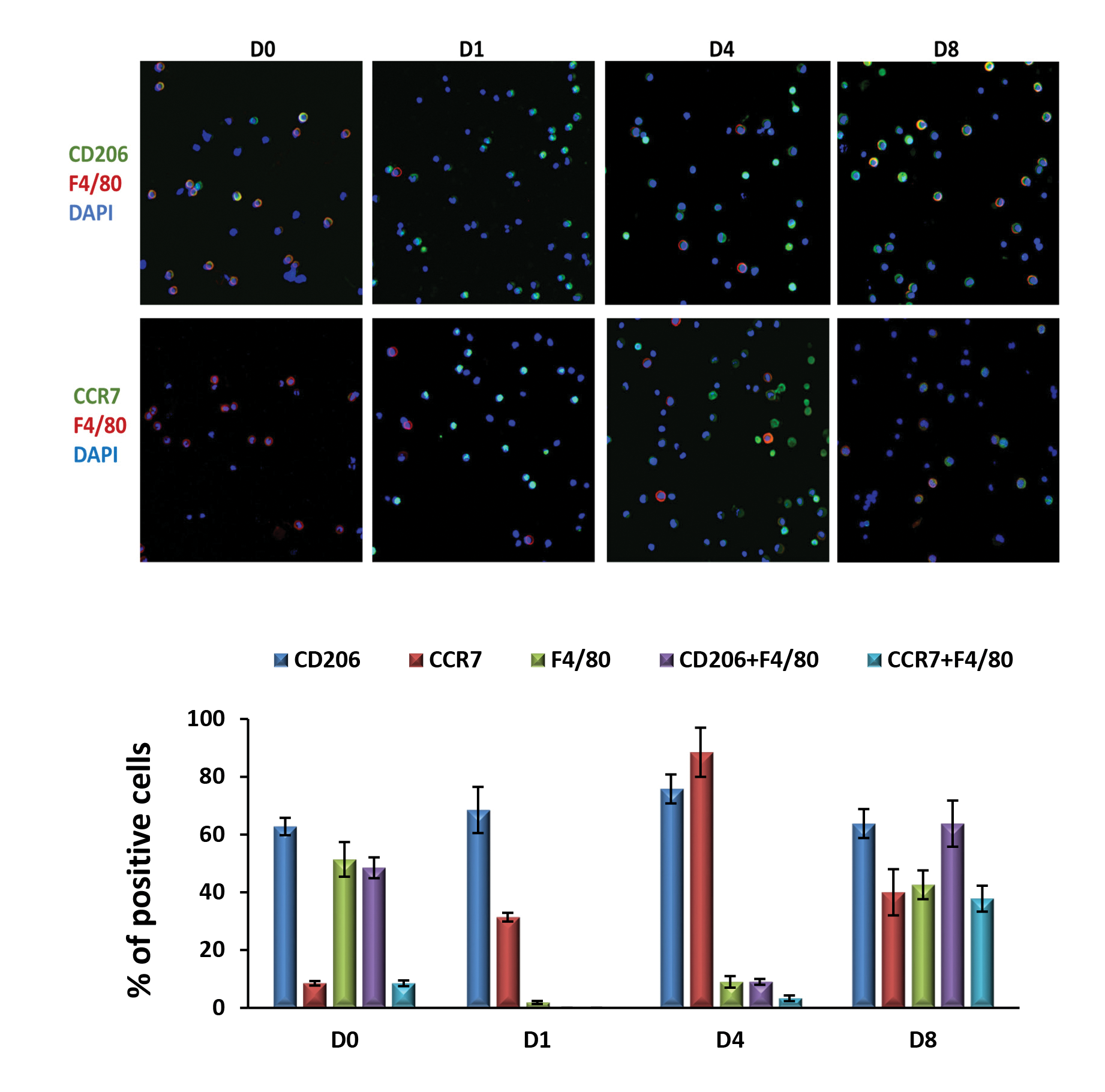

Supplement: S1 Fig — Endometriosis was induced in immunocompetent host females and maintained with E2. Peritoneal cells were harvested at the indicated time points and subjected to double Immunofluorescent labeling for CD206 or CCR7 with F4/80, respectively. Representative images at each time point are shown (top panels, 20X). The percentages of positive cell numbers for CD206, CCR7, CCR7+/F4/80+, or CD206+/F4/80+ are shown (bottom panel). The numerical values were analyzed by One-way ANOVA followed by Dunnett’s post hoc test and expressed as mean ± SEM (n = 5). *p < 0.01 verse D0. (TIF) [file pone.0165347.s001.tif]

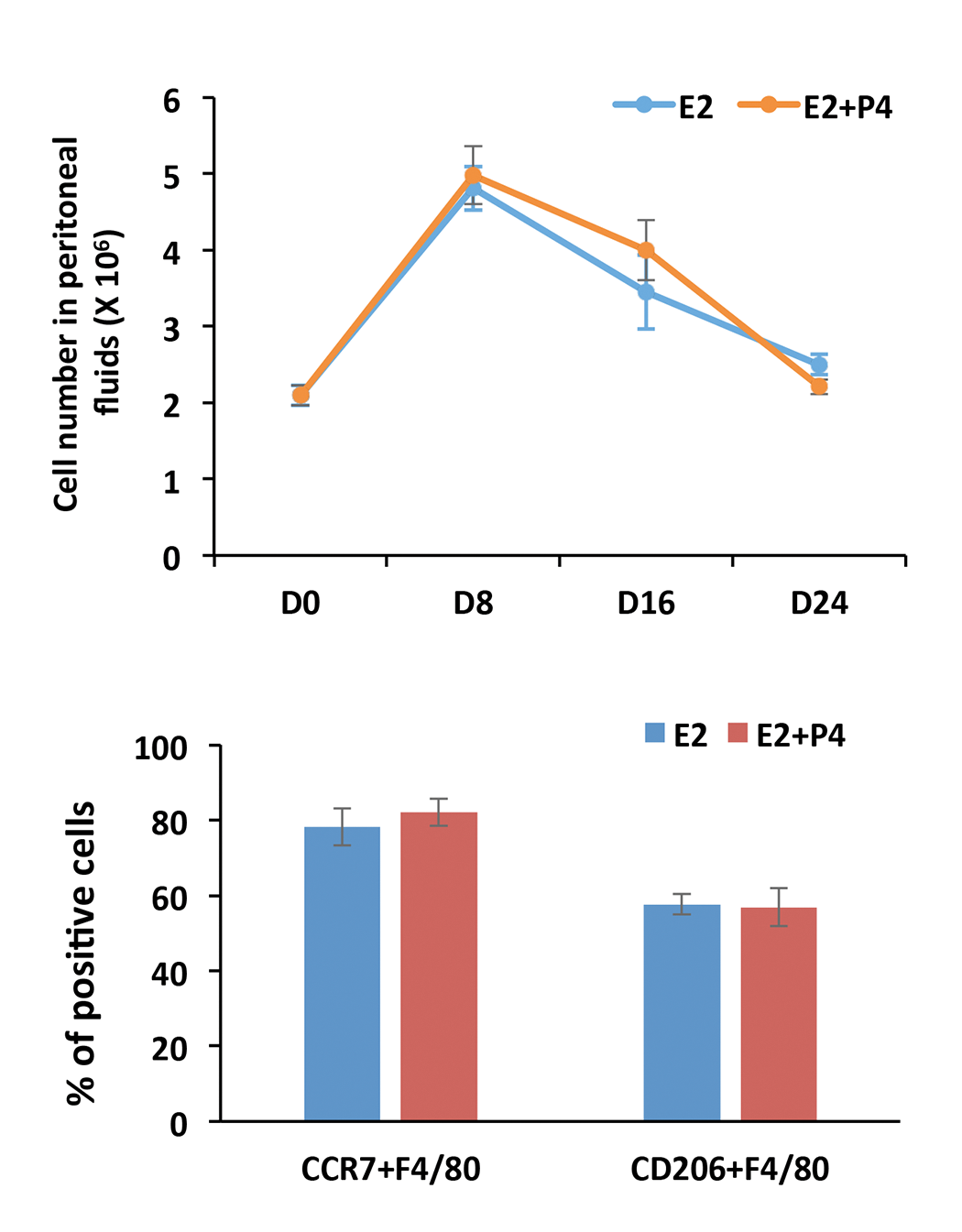

Supplement: S2 Fig — Ectopic lesions were induced in E2-, or E2 plus P4-treated immunocompetent host females. The total number of exuded peritoneal cells were counted (A) and subjected to double immunofluorescent labeling for CD206 or CCR7 with F4/80 (B). The average percentages of cell numbers for CCR7+/F4/80+ or CD206+/F4/80+ are shown. The numerical values were analyzed by One-way ANOVA followed by Dunnett’s post hoc test and expressed as mean ± SEM (n = 5). Statistical significance is defined as #: p < 0.05, *: p<0.01 verse D0. (TIF) [file pone.0165347.s002.tif]

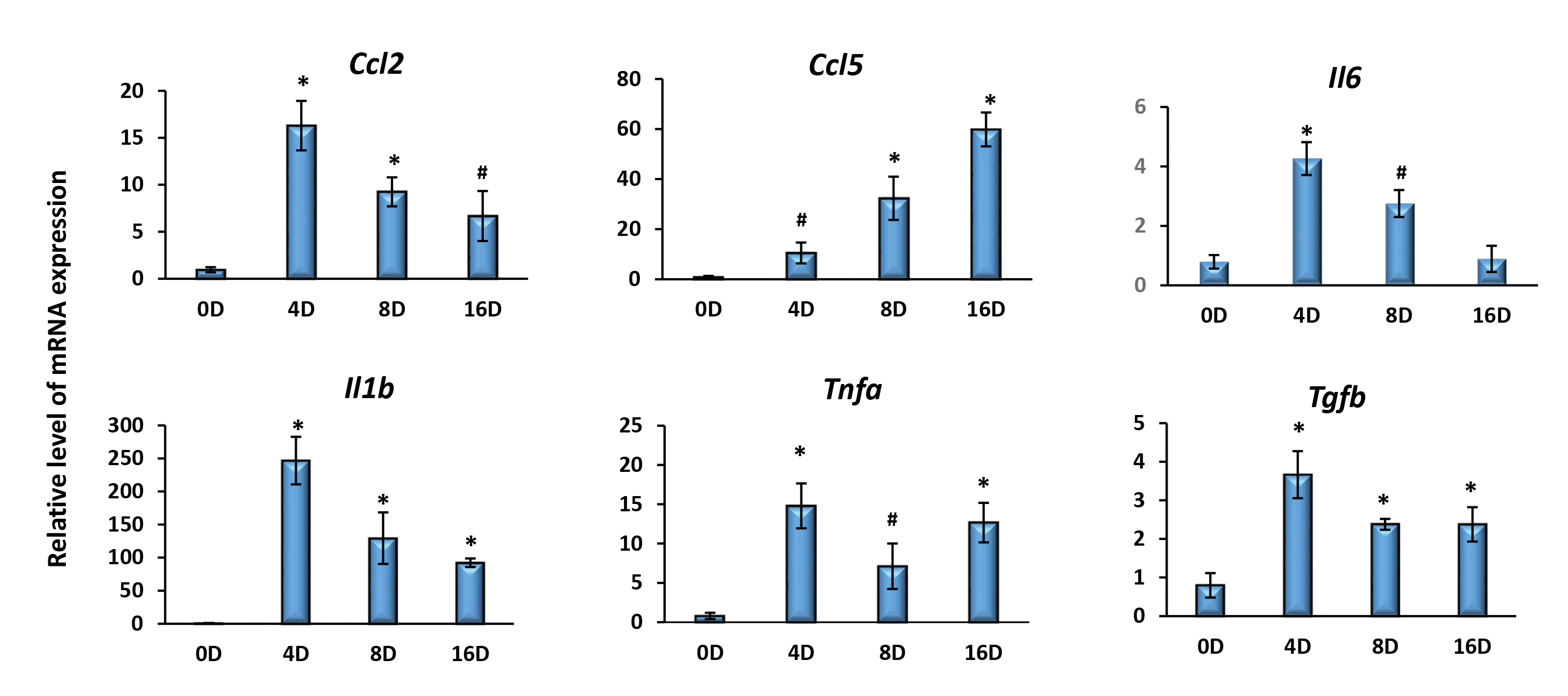

Supplement: S3 Fig — Endometriosis was induced in immunocompetent female mice and maintained with E2. The ectopic lesions were harvested on days 0, 4, 8, and 16 after induction and subjected to qPCR analysis to assess the relative level of gene expression corresponding to Ccl2, Ccl5, Il1b, Il6, Tnfα, and Tgfβ, respectively. The numerical values were analyzed by One-way ANOVA followed by Dunnett’s post hoc test and expressed as mean ± SEM (n = 6). Statistical significance is defined as #: p < 0.05, *: p<0.01 verse D0. (TIF) [file pone.0165347.s003.tif]

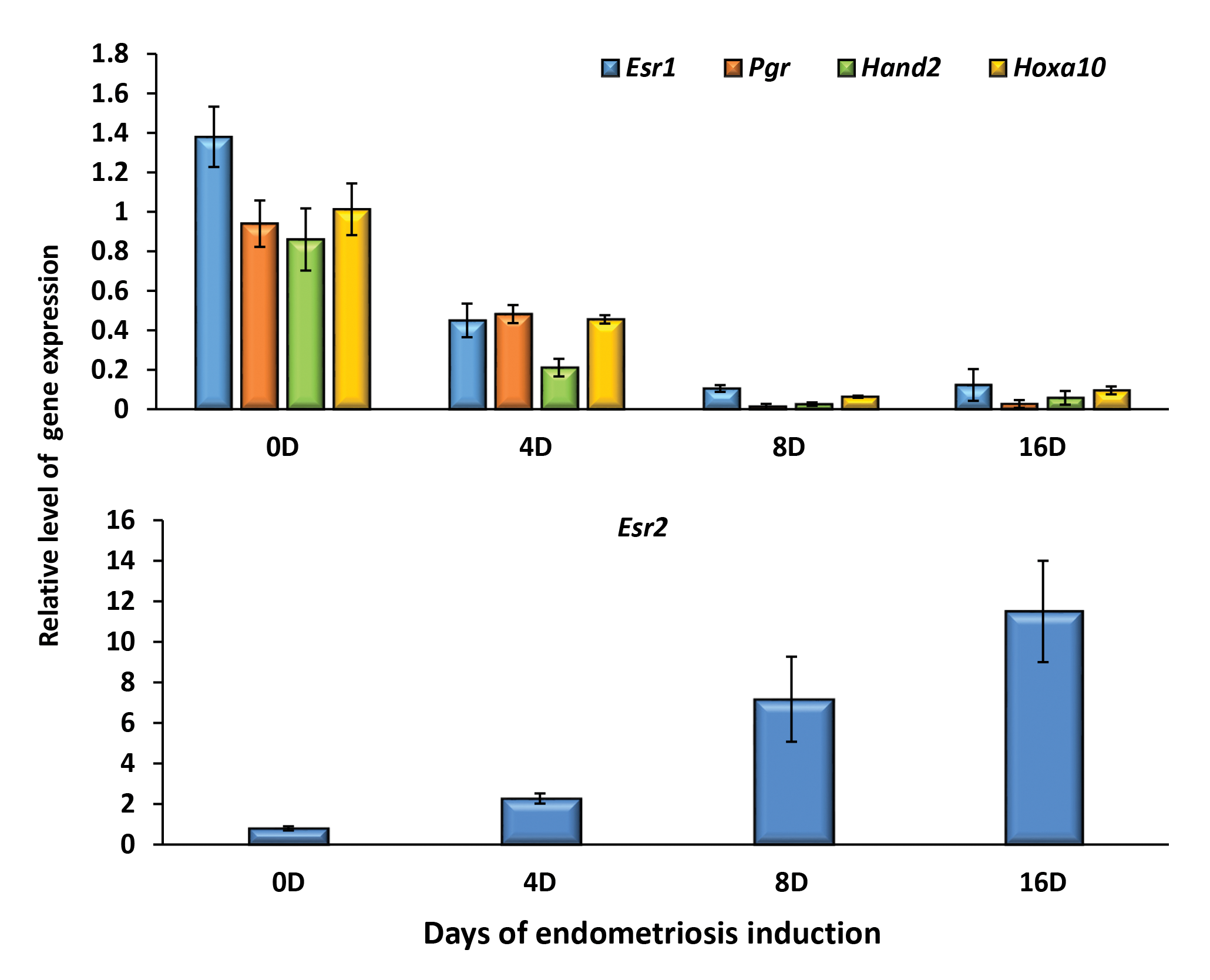

Supplement: S4 Fig — Endometriosis was induced in immunocompetent female mice and maintained with E2. The ectopic lesions were harvested on Days 0, 4, 8, and 16 after induction (N = 6) and subjected to qPCR analysis to assess the relative level of gene expression corresponding to Esr1, Pgr, Hand2, and Hoxa10 (upper panel), or Esr2 (Lower panel), respectively. The numerical values were analyzed by One-way ANOVA followed by Dunnett’s post hoc test and expressed as mean ± SEM. Statistical significance is defined as #: p < 0.05, *: p<0.01 verse D0. (TIF) [file pone.0165347.s004.tif]
