# Supplementary material for: Progesterone Alleviates Endometriosis via Inhibition of Uterine Cell Proliferation, Inflammation and Angiogenesis in an Immunocompetent Mouse Model
Source: PLoS One. 2016 Oct 24;11(10):e0165347. doi: 10.1371/journal.pone.0165347 (PMC5077092; doi:10.1371/journal.pone.0165347)
Supplement: S1 File — (PDF) [file pone.0165347.s005.pdf]

Supplemental Table A: Antibodies used in this study

**Peptide/protein target**	**Name of Antibody**	**Manufacturer, catalog #,**	**Species raised in; monoclonal or polyclonal**	**Dilution used**
alpha smooth muscle actin	αSMA	Abcam, ab5694	Rabbit, polyclonal	200
Cysteine-rich angiogenic inducer 61	Cyr61/CCN1	Novus Biologicals NB100-356	Rabbit, polyclonal	100
C-C Motif Chemokine Receptor 7	CCR7	Abcam, ab103404	Rabbit, polyclonal	200
mannose receptor	CD206	Abcam, ab64693	rabbit, polyclonal	200
Platelet endothelial cell adhesion molecule	PECAM-1/CD31	Abcam, ab28364	rabbit, polyclonal	200
Cluster of Differentiation 68	CD68	Abcam, ab955	Rabbit, polyclonal	200
Cytokeratin 11	CK11	Developmental Studies Hybridoma Bank, TROMA-I-s	Rat, monoclonal	50
Estrogen Receptor-a	ERα	Novocastra, NCL-L-ER 6F11	mouse, monoclonal	200
EGF-like module-containing mucin-like hormone receptor-like 1	EMR1/F4/80	eBioscience,12-4801	Rat, monoclonal	300
Forkhead box P3	FoxP3	ab20034	mouse, monoclonal	10 ug/ml
Heart And Neural Crest Derivatives Expressed 2	HAND2/dHand	Santa Cruz, sc-9409 (m-19)	goat, polyclonal	200
ki67	KI67	BD Pharmingen, 550609	mouse, monoclonal	250
Progesterone Receptor	PR	Dako, A0098	rabbit, polyclonal	100
RAR-related orphan receptor gamma	RORγ(t),	eBioscience, 14-6988	Rat, monoclonal	5 ug/ml
Vimentin	VIM	Cell Signaling, 5714S		
Alexa Fluor® 488 AffiniPure Donkey Anti-Rabbit IgG (H+L)		Jackson ImmunoResearch Laboratories, 711-545-152;	Donkey ,polyclonal	200
Rhodamine Red™-X (RRX) AffiniPure Donkey Anti-Mouse IgG (H+L)		Jackson ImmunoResearch Laboratories, 715-295-150	Donkey ,polyclonal	200

Supplemental Table B: Primers Used for qPCR Analyses

Gene	Sequence	Amplicon Size (bp)
Esr1	For: CCTCCCGCCTTCTACAGGT<br>Rev: CACACGGCACAGTAGCGAG	128
Pgr	For: CTCGGACGTGTCGTCTGTAG<br>Rev: CCTGTCTTTCCGTCTGGGAG	102
Hand2	For: CATAATGGGAGTGGTCCAG<br>Rev: TACTTCCACGGCTGGCTTAT	118
Hoxa10	For: GGAAGCATGGACATTCAGGT<br>Rev: CCAGGCAAGCAAGACCTTAG	182
Ccl2	For: TTAAAAACCTGGATCGGAACCAA<br>Rev: GCATTAGCTTCAGATTTACGGGT	121
Ccl5	For: GCTGCTTTGCCTACCTCTCC<br>Rev: TCGAGTGACAAACACGACTGC	104
Il1b	For: GCAACTGTTCCTGAACTCAACT<br>Rev: ATCTTTTGGGGTCCGTCAACT	89
Il6	For: TAGTCCTTCCTACCCCAATTTCC<br>Rev: TTGGTCCTTAGCCACTCCTTC	76
Tnfa	For: CCCTCACACTCAGATCATCTTCT<br>Rev: GCTACGACGTGGGCTACAG	61
Tgfb	For: CCACCTGCAAGACCATCGAC<br>Rev: CTGGCGAGCCTTAGTTTGGAC	91
Ptgs2	For: TGAGCAACTATTCCAAACCAGC<br>Rev: GCACGTAGTCTTCGATCACTATC	74
36B4	For: CATCACCACGAAAATCTCCA<br>Rev: TTGTCAAACACCTGCTGGAT	159

for, forward primer; rev, reverse primer; bp, base pairs.
